# Sensitivity Analysis of Sidelobes of the Lowest Order Cladding Mode of Long Period Fiber Gratings at Turn Around Point

**DOI:** 10.3390/s22082965

**Published:** 2022-04-12

**Authors:** Tanoy Kumar Dey, Sara Tombelli, Arpan Roy, Palas Biswas, Ambra Giannetti, Nandini Basumallick, Francesco Baldini, Somnath Bandyopadhyay, Cosimo Trono

**Affiliations:** 1 Central Glass and Ceramic Research Institute, CSIR-CGCRI, 196 Raja S C Mullick Road, Kolkata 700032, India; tanoykumardey@gmail.com (T.K.D.); roy.arpan456@gmail.com (A.R.); palas@cgcri.res.in (P.B.); nandini_b@cgcri.res.in (N.B.); 2 Institute of Applied Physics “Nello Carrara”, CNR-IFAC, Via Madonna del Piano 10, 50019 Sesto Fiorentino, Italy; s.tombelli@ifac.cnr.it (S.T.); a.giannetti@ifac.cnr.it (A.G.); f.baldini@ifac.cnr.it (F.B.)

**Keywords:** long period fiber grating, etching, cladding mode, sidelobes, sensitivity enhancement

## Abstract

A new methodology to enhance the sensitivity of a long period fiber grating sensor (LPFG) at the Turn Around Point (TAP) is here presented. The LPFG sensor has been fabricated by etching the fiber up to 20.4 µm, until the sidelobes of dispersed LP_0,2_ cladding mode appeared near TAP in aqueous medium. The dual peak sensitivity of the sidelobes was found to be 16,044 nm/SRIU (surrounding refractive index units) in the RI range from 1.333 to 1.3335.

## 1. Introduction

Long period fiber gratings (LPFGs) have been widely used as biochemical sensors due to their ability of surrounding refractive index (SRI) sensing [1]. In most biochemical sensing applications, the SRI remains around 1.333 [2,3] and, in this region, the SRI sensitivity of conventional LPFG sensors is not sufficiently high [4]. To overcome this problem, different methodologies have been adopted to enhance the sensitivity of the sensor in this region, which are broadly classified in three categories. (1) working around mode transition (MT) of a desired cladding mode (CM) [2,5,6,7,8], (2) working near Turn Around Point (TAP) of a CM [9,10] and (3) enhancement of the evanescent field of the sensor [9,11,12,13]. Combinations of these three methodologies have also been reported [14,15,16,17,18,19]. For the first methodology, different overlay deposition techniques are required to make the LPFG work in MT: some of them are critical and some are expensive [2,17,18,19]. On the contrary, the other two methodologies are rather easy to attain by controlling the grating period and reducing the cladding diameter [13,15]. Recently, SRI sensitivity of the LPFG sensor has been optimized by combining the effect of highest enhancement of the evanescent field and working near TAP of a dispersed CM [13]. Some innovative configurations of the LPFG have also been proposed as highly sensitive RI sensors and chemical sensors [20,21].

In the context of LPFG sensors, sidelobes of CMs have been not considered yet: these are basically the result of constructive and destructive interference between core mode and CM [22] and they are always treated as noise in the domain of sensor and tried to be suppressed by adopting different techniques [23]. Only a few works made an attempt to explain these sidelobes analytically, but not for sensing purposes [24].

In this paper, we introduce for the first time, to the best of our knowledge, the sidelobes of the CM as a new methodology to enhance the sensitivity of an LPFG sensor. The sensor was fabricated by combining this effect with maximum enhancement of evanescent field and working around TAP. The cladding diameter was reduced by chemical etching until the sidelobes of dispersed LP_0,2_ CM appeared near TAP in aqueous medium. The sensor was characterized in term of SRI sensitivity which was found out to be 16,044 nm/SRIU (surrounding refractive index unit), ~1.8 times higher than the sensitivity of LP_0,2_ CM near TAP [13].

## 2. Materials and Methods

### 2.1. LPFG Fabrication and Fiber Etching

The LPFG was fabricated on Fibercore PS 1250/1500 B/Ge co-doped photosensitive fiber (cutoff wavelength 1209 nm), using point by point inscription technique with a KrF excimer laser (Compex 110, Lambda Physics GmbH, Gottingen, Germany). The grating period 𝛬 was 246 µm and the number of grating planes was 123. The period and number of grating planes were chosen in order to maintain the TAP of LP_0,2_ CM in C + L band after cladding diameter reduction [13]. A micrometer slit with 50% duty cycle was used to shape the laser beam during inscription and cylindrical lens was used to focus the beam along the axis of the fiber. Total pulse per plane was 80 and total fluence per plane was 22.4 J/cm^2^. The stripping length of the fiber was 40 mm and the grating was inscribed in the middle portion of the naked fiber region. For etching, the fiber was mounted straight on a u-shaped holder and dipped in 20% hydrofluoric acid (HF) (Merck, Milan, Italy) in water to reduce the cladding diameter. The etching process was continued until the dispersed LP_0,2_ CM appeared within the bandwidth of interest [13].

### 2.2. Data Acquisition and Data Analysis

The used optical source was the SLED SLD-1310/1430/1550/1690-10 (FiberLabs Inc., Saitama, Japan). All spectra were measured using an optical spectrum analyzer (OSA) MS9030A/9701C (Anritsu, Kanagawa, Japan).

The minimum wavelengths were calculated by fitting the resonant band with a Lorentzian function. The fitting operation was performed on a subset of the spectrum data in a 20 nm wavelength band around the minimum wavelength, following the procedure described in the Supporting Information of [6].

### 2.3. Refractometric Sensitivity Analysis

For the refractometric sensitivity analysis a set of 6 sodium chloride (NaCl) (Merck, Milan, Italy) solutions in water in the range 0.0% to 0.5% (1.333 to 1.3335 RI range) was used.

## 3. Results and Discussion

### 3.1. Etching of Fiber, TAP of the LP_0,2_

**Figure 1:**
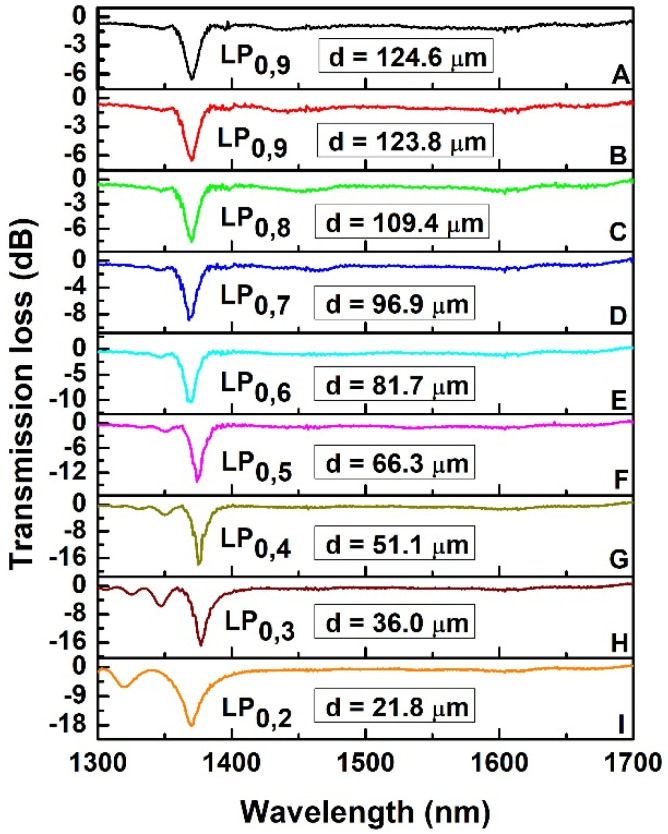
Spectral evolution of LP_0,x_ CM (x from 9 to 2) at ~1370 nm by reduction of cladding diameter. ((**A**): LP_0,9_ CM after inscription; (**B**): LP_0,9_ CM with cladding diameter 123.8 µm; (**C**): LP_0,8_ CM with cladding diameter 109.4 µm; (**D**): LP_0,7_ CM with cladding diameter 96.9 µm; (**E**): LP_0,6_ CM with cladding diameter 81.7 µm; (**F**): LP_0,5_ CM with cladding diameter 66.3 µm; (**G**): LP_0,4_ CM with cladding diameter 51.1 µm; (**H**): LP_0,3_ CM with cladding diameter 36.0 µm; (**I**): LP_0,2_ CM with cladding diameter 21.8 µm).

The spectrum of the LPFG at the end of the inscription process and before etching is shown in Figure 1A, where the left peak of the LP_0,9_ CM appears at ~1370 nm with transmission loss (TL) of −6.5 dB. The considered bandwidth was from 1300 nm to 1700 nm, depending on the available SLED source and the cutoff wavelength (1209 nm) of the fiber.

The spectral evolution during etching is shown in Figure 1B–I, where the spectra of different CMs are shown when the left peak is at ~1370 nm for a better comparison. The right peaks of the CMs are not visible within the bandwidth as their resonant wavelength was higher than 1700 nm, and so out of OSA bandwidth. The corresponding diameters of the fiber when the left peak is at 1370 nm are also calculated and shown in the Figure 1.

**Figure 2:**
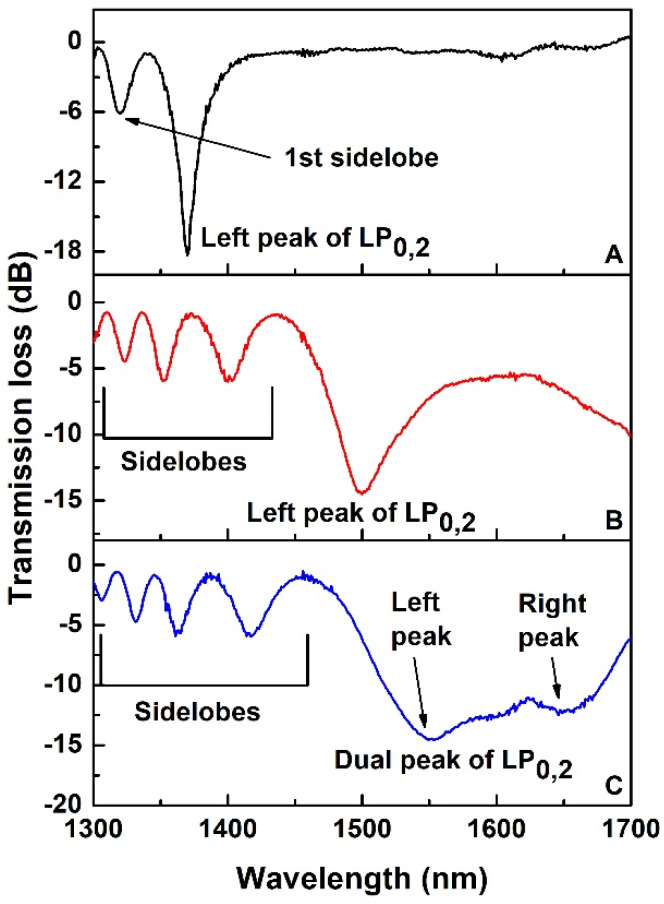
Spectral evolution of LP_0,2_ CM near TAP in aqueous medium. ((**A**): Left peak of LP_0,2_ CM at ~1370 nm; (**B**): Left peak of LP_0,2_ CM at ~1500 nm; (**C**): Dual peak of LP_0,2_ CM near TAP).

**Figure 3:**
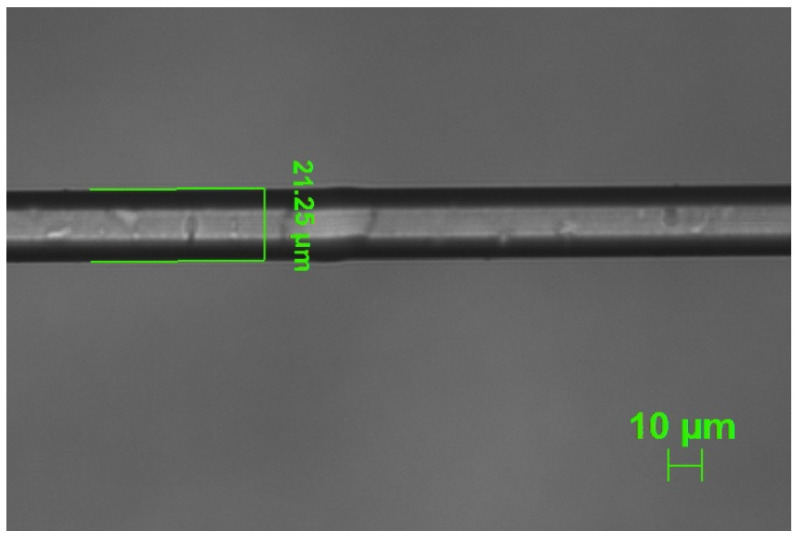
Microscopic image of etched fiber with 20× magnification.

It is clear from the Figure 1 that the sidelobes of CM were becoming prominent as the cladding diameter was reduced. They started to become clearly visible when LP_0,5_ dispersed CM appeared within the bandwidth (Figure 1F) with their TL increasing as consecutive lower order dispersed CMs appeared as a consequence of the reduction of cladding diameter: the sidelobe TL was maximum (~−6.2 dB) when the LP_0,2_ dispersed CM appeared within the bandwidth. The etching process was further continued to bring the LP_0,2_ dispersed CM near TAP. At this point, both the left and right peaks of the LP_0,2_ cladding mode at TAP were visible, being within the OSA bandwidth, with peak-to-peak separation of 110 nm (left peak at ~1550 nm, right peak at ~1660 nm). The detailed spectral evolution that highlights the appearance of the right peak of the LP_0,2_ cladding mode at around 1660 nm as well as of the left sidelobes of the left peak is shown in Figure 2, where, for the sake of clarity, the first spectrum (spectrum A) is the same of the last spectrum in Figure 1 (spectrum I). In this case, the spectra were acquired by pulling out the fiber from the HF solution and placing it in water. The measured diameter of the fiber at this point was ~21.25 µm as shown in Figure 3.

### 3.2. Etching of Fiber, Side Lobes of the LP_0,2_

After bringing the dual peaks of LP_0,2_ CM near TAP, the sensor was put in a closed thermostated flow cell [13]. This is an essential step for three reasons: (i) the easy handle of a very low diameter fiber; (ii) the reduction of the temperature, strain and bending related cross-sensitivities; (iii) the precise and low-noise control of all the fluidic procedures.

The sensitivity of LP_0,2_ CM was measured by using 0.0% to 0.5% *w*/*v* NaCl water solutions (RI in the range 1.333 to 1.3335). The dual peak sensitivity was found out to be 8728 nm/SRIU [13]. The dimensions of the flow cell, the process of positioning the fiber into the cell and sensitivity measurement procedure were described in our previous work [13].

**Figure 4:**
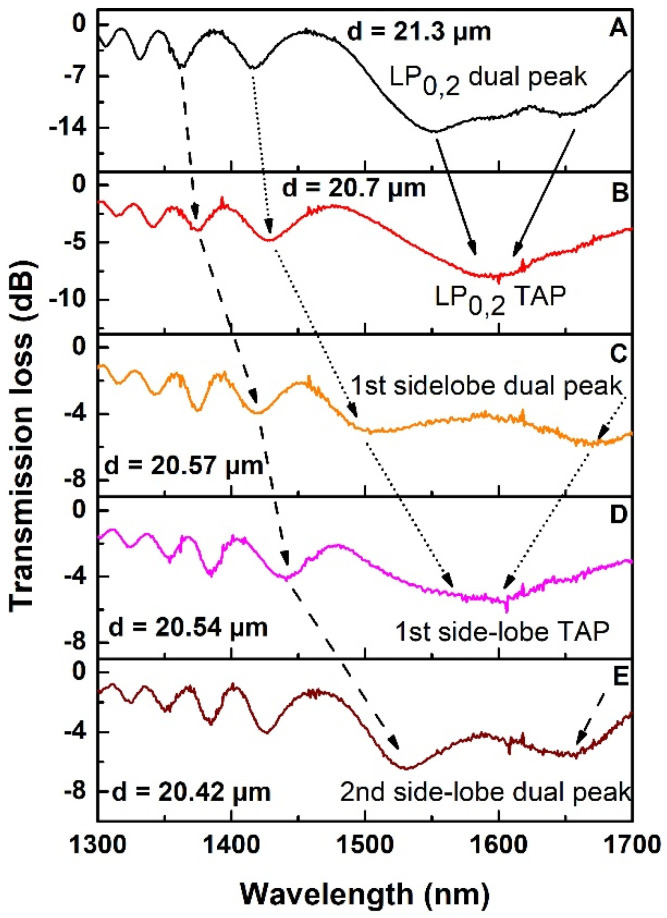
Spectral evolution of sidelobes of LP_0,2_ CM at TAP. ((**A**): Dual peak of LP_0,2_ CM; (**B**): TAP of LP_0,2_ CM; (**C**): 1st side-lobe dual peak; (**D**): 1st side-lobe TAP; (**E**): 2nd sidelobe dual-peak).

After sensitivity analysis, the flow channel was cleaned with water and 1% HF was inserted into the channel to further reduce the cladding diameter. The concentration of the HF solution was reduced to slow down the etching rate, so that the spectral position can be precisely controlled. As a result of the cladding reduction from 21.3 µm to 20.42 µm, a shift of the CM peaks and of the sidelobes takes place, as shown in Figure 4 which reports the sequence of the spectral evolution:

- the dual peak of LP_0,2_ CM merged together at TAP and single peak is generated at about 1600 nm (Figure 4B);
- the merged CM peak decreases and disappears while the first right sidelobe appears for the first time within the OSA window (Figure 4C);
- the dual peaks of the first sidelobes merge in a single peak at TAP (Figure 4D);
- the merged sidelobe peak decreases and disappears while also the second right sidelobe appears Figure 4E).

The arrows in Figure 4 highlight this spectral evolution: solid, dotted and dashed arrows follow the evolution of CM, first sidelobes and second sidelobes, respectively. In Figure 4, the calculated values of the fiber diameter are also reported. At this point, the etching process was concluded because further etching could bring at the TAP higher order sidelobes with lower attenuation depth and broader shape with a consequent increase of the noise in the determination of the minimum wavelength and the worsening of the limit of detection (LOD). It is worth to note that, starting from the conditions of Figure 4C, the sensor is working beyond the TAP of LP_0,2_ CM in aqueous solution.

### 3.3. Theoretical Discussion and Modeling

The reduction of the cladding diameter directly influences the coupling coefficient (*k*) of CMs [13]. The etching process started with LP_0,9_ CM in under-coupled condition (*kL* < π/2, where *L*= length of the grating), and the coupling coefficient was increased with the etching process, as demonstrated by the increase of TL of the dispersed CMs, shown in Figure 1. The coupling coefficient of the CMs does also influence its corresponding sidelobes [24]. As the LP_0,9_ CM was in under-coupled condition, the sidelobes were not visible in that spectrum (Figure 1A). However, they became visible with lower order dispersed CMs (after LP_0,5_ CM), which were the result of cladding diameter reduction.

The TL at any wavelength of the LPFG spectrum can be calculated using the following Equation (1):

(1) $$T=\cos^{2}\left[l\sqrt{\delta^{2}+k^{2}}\right]+\frac{\delta^{2}}{\delta^{2}+k^{2}}\sin^{2}\left[l\sqrt{\delta^{2}+k^{2}}\right]$$

which shows that for a fixed length of the grating, the TL depends on the detuning parameter (*δ*), and the coupling coefficient (*k*). The simulations of the phase matching curves (PMCs) of CMs demonstrate that the cladding diameter reduction also influences their shape. The fiber grating parameters considered for the simulation of the PMCs were: core RI n1 = 1.44985; cladding RI n2 = 1.44400; core diameter: 7.3 µm.

**Figure 5:**
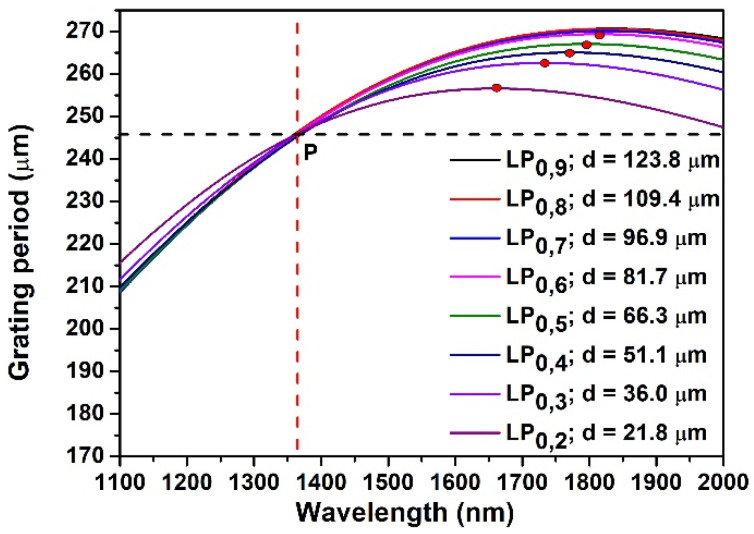
PMCs of LP_0,9_ to LP_0,2_ CMs at different diameter in SRI 1.333.

For an easier comparison of different PMCs, the PMCs in SRI 1.333 were calculated considering the diameters related to the respective CMs as mentioned in Figure 1, so that the PMCs can all intersect at a single point (P) where the grating period was 246 µm and resonant wavelength was 1370 nm, as shown in Figure 5. It is clear from Figure 5 that the shape of PMCs of LP_0,9_, LP_0,8_, LP_0,7_ and LP_0,6_ did not significantly change as a consequence of the reduction of cladding diameter, but this change was prominent starting from LP_0,5_, when also sidelobes become prominent. As the cladding diameter was reduced, the TAP of lower order CMs (red dot) moved toward blue direction and its position was shifted downward. As a result, the point P was becoming closer to the TAP, and the slopes of the PMCs at point P were reduced, i.e., the PMCs began flattening (Table 1). By looking at the PMC flatness, indicated by the slope values reported in Table 1, a relation can be observed between the cladding diameter reduction together with the PMC flatness, and the appearance of sidelobes, which started to be prominent and observable when the slopes cross the cutoff value of 106.

**Figure 6:**
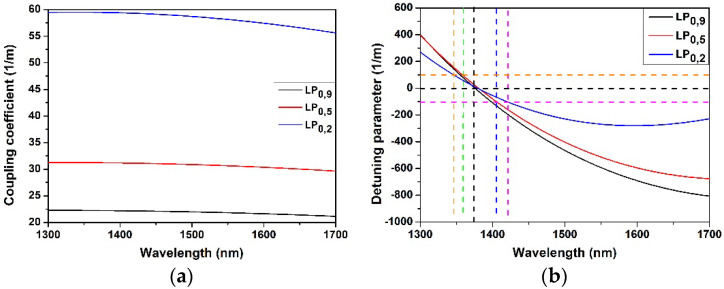
Coupling coefficients (**a**) and detuning parameter (**b**) of LP_0,9_, LP_0,5_ and LP_0,2_ CMs at different wavelength.

We also computed the coupling coefficients (*k*) and the detuning factor (*δ*) of LP_0,9_ CM and two dispersed CMs, LP_0,5_ and LP_0,2_ with reduced cladding radii as a function of wavelength. The computed results are shown in Figure 6a,b, respectively. From Figure 6a, it is apparent that the coupling coefficient of the dispersed modes (LP_0,5_ and LP_0,2_ in this case) is much higher than the coupling coefficient of the LP_0,9_ mode which justifies the experimentally observed increase of the attenuation of the resonances. 

The spectral evolution reported in Figure 1 shows also the increase of the mutual spectral distance between the cladding mode peak and the associated sidelobes, as reported in Table 2. This phenomenon can be explained with the results of the modelling, shown in Figure 6b. The dashed vertical line in black designates the resonant wavelength of the CMs at ~1370 nm where the detuning factor is zero, namely the central peak. Two dashed horizontal lines defines a detuning *δ* = ±100. Intersection of these two lines with the detuning curves of individual CMs defines the resonant wavelength of the sidelobes with this specific detuning (indicated with dashed vertical lines). It may be noted that the slope of the detuning curves decreases with the mode dispersion. This causes unequal positioning of the sidebands having equal detuning with respect to the central lobe, as shown in Figure 6b.

The results of the simulations showed that, with reduction of cladding diameter: (i) the coupling coefficient decreases as a function of wavelength for a specific mode (Figure 6a); (ii) the TAP wavelength shifts in red direction (Figure 5); (iii) the phase matching curve (Figure 5) and the detuning parameter curve (Figure 6b) become flatter. This last effect results in larger separation of the theoretically expected right sidelobes. Moreover, the PMCs are no longer monotonic, with a minimum value in the spectral region where right sidelobes should appear. All these effects could contribute to the noticeably lower strength of the sidelobes between the two peaks at TAP. So experimentally we see the sidelobes on the right of the left CM peak are significantly weaker (practically not visible) than the sidelobes on the left (Figure 1 and Figure 2). This should be also true for the right peak of the CM, where the sidelobes should appear only at the right side of the resonant peaks but in a spectral band that is out of the working range of the OSA and of the source.

### 3.4. Sensitiviy Analysis

**Figure 7:**
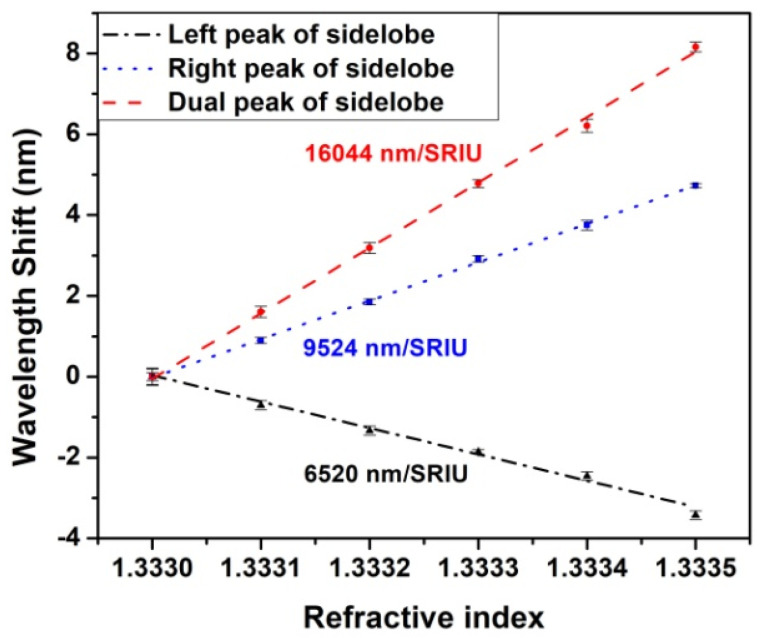
Sensitivity analysis of 2nd sidelobes of LP_0,2_ CM at TAP.

The sensitivity of the 2nd sidelobe dual peak was measured in the 1.333 to 1.3335 RI range obtaining the values of 6520 nm/SRIU, 9524 nm/SRIU and 16,044 nm/SRIU for the left, right and dual peak (shown in Figure 7), respectively, leading to a value 1.8 times better than working with dual peaks of LP_0,2_ CM near TAP (8751 nm/SRIU, [13]). In general, in case of LPFG resonances near the TAP, together with the sensitivity enhancement there is a worsening of the shape of the resonance spectra, characterized in particular by the increase of the resonance bandwidth (see for example the spectra reported in Figure 2). For this reason, the minimum wavelength was calculated by means of a fit of the experimental data with a Lorentzian function that allows to reduce the uncertainty in its determination. The error for each tested RI, computed as the root sum square of the standard deviations of the 8 measurements acquired (the results of the fit operation on left and right peaks of 8 acquired spectra) during the stop flow phase in the thermostated flow-cell for 3 min, is in the 0.1–0.2 nm range (0.14 nm mean value). The standard deviation calculated on a long-term acquisition (1 h in flow cell and stop flow condition) was 0.2 nm. The resolution, calculated as three times the average standard deviation divided by the sensitivity is 2.6 × 10^−5^ SRIU.

It is worth to mention here that, in general, for the dual peak sensing mechanism, the sensitivity depends greatly on the separation of these two resonance peaks and increases when the separation decreases. In the present case, the dual peak sidelobes sensitivity was calculated when the separation was 130 nm while in [13] the separation of the LP_0,2_ cladding mode dual peak was 104 nm, so it can be expected that, in the same conditions, the sidelobes sensitivity will be even better. Moreover, we suppose that the sensitivity should increase with fiber diameter reduction, which generates higher order sidelobes near TAP. We considered here the 2nd sidelobes since further etching could cause lower attenuation depth and broader shape of the higher sidelobes, worsening the LOD.

## 4. Conclusions

A LPFG sensor was fabricated with novel sensitivity enhancement technique by introducing the sidelobes of the CM, along with maximum enhancement of the evanescent field and working near TAP in aqueous medium. Due to the introduction of the sidelobes near the TAP, it became possible to work beyond the TAP of the “conventional” cladding mode, obtaining a dual peak sensitivity of 16,044 nm/RIU. The high sensitivity of the sensor near SRI 1.333 and the mechanical and thermal stability obtained with the microfluidic cell, made the sensor suitable for high resolution biosensing application.

## Figures and Tables

**Table 1:** Simulated fiber diameters and slope at point P for different order of CMs.

Cladding Modes	Fiber Diameter (µm)	Slope at Point P
LP_0,9_	123.8	106
LP_0,8_	109.4	106
LP_0,7_	96.9	106
LP_0,6_	81.7	106
LP_0,5_	66.3	103
LP_0,4_	51.1	99
LP_0,3_	36.0	92
LP_0,2_	21.8	74

**Table 2:** Side lobes wavelength relative to the mean peak.

	Normalized Main Peak Spectral Position	1st Sidelobe (nm)	2nd Sidelobe (nm)	3rd Sidelobe (nm)
LP_0,5_	0	24.22	Not visible	Not visible
LP_0,4_	0	24.22	44.44	Not visible
LP_0,3_	0	27.66	50.78	69.22
LP_0,2_	0	55.33	Out of OSA	Out of OSA

## Data Availability

Not applicable.
